# Socioeconomic status and barriers for contacting the general practitioner when bothered by erectile dysfunction: a population-based cross-sectional study

**DOI:** 10.1186/s12875-020-01238-2

**Published:** 2020-08-16

**Authors:** Sanne Rasmussen, Kirubakaran Balasubramaniam, Dorte Ejg Jarbøl, Jens Søndergaard, Peter Fentz Haastrup

**Affiliations:** Research Unit of General Practice, Department of Public Health, University of Southern Denmark, J.B. Winsløws Vej 9A, 5000 Odense C, Denmark

**Keywords:** Erectile dysfunction, Healthcare seeking, Socioeconomic characteristics, Barriers

## Abstract

**Background:**

Erectile dysfunction (ED) is common and impacts psychosocial wellbeing negatively. Many do not seek medical attention and several barriers for healthcare seeking with ED exist. Little is known about the association between socioeconomic characteristics of the patient and barriers for healthcare-seeking for men bothered by ED.

The objectives of the study were 1) to estimate the proportion of men bothered by ED, who do not contact the GP, 2) to analyse the frequencies of selected barriers for healthcare seeking and 3) to analyse associations between socioeconomic factors and barriers for contacting the GP.

**Methods:**

Data derive from a nationwide survey of symptom experiences among 100,000 randomly selected individuals aged 20 years and above. The questionnaire comprises, among other, questions about ED. This study focuses on men who reported bothersome ED and further reported, that they did not contact a GP regarding the symptom. Questions addressing barriers regarding GP contact included embarrassment, worrying about wasting the doctor’s time, being too busy, and worrying about what the doctor might find. Information about socioeconomic characteristics was obtained from Statistics Denmark.

**Results:**

A total of 4072 men (18.3%) reported that they had experienced ED within the past four weeks. Of those, 2888 (70.9%) were categorized as having bothersome ED. In the group of men with bothersome ED 1802 (62.4%) did not contact the GP and 60.5% reported barriers for GP-contact. Of the reported barriers, the most frequent was ‘being too embarrassed’ (29.7%). In general, respondents in the older age groups were less likely to report embarrassment, business and worrying what the doctor might find. Respondents with highest attained educational level were less likely to report embarrassment and worrying.

**Conclusion:**

Nearly two third of the respondents with bothersome ED had not contacted their GP. More than half of those reported barriers towards GP contact with embarrassment as the most frequent barrier. In general, respondents in the older age groups and with high educational level were less likely to report barriers.

## Background

Erectile dysfunction (ED) refers to difficulties to achieve or maintain penile erection sufficient to complete sexual activities satisfactorily. The diagnosis is therefore based on the patient’s reported symptoms of erection difficulties and objective physiological measures of erectile function are usually not part of the diagnostic process [1]. ED does not only cause sexual difficulties but also diminished confidence and low self-esteem [2].

ED is common among both younger and older men in various settings [3, 4] and is estimated to be experienced by 19.3% of the population [5] with higher incidence with increasing age [6].

If presented to the healthcare system, diagnosis and treatment of ED is most often handled by the general practitioner (GP) [7] and effective treatment options are available, improving erectile function and the psychosocial outcomes associated with ED [2]. However, a prerequisite for diagnosis and treatment is that men contact their GP regarding ED. The process of healthcare seeking in general is influenced by a mixture of physical, social and psychological factors [8] where some factors are drivers and other are barriers. It has been shown that most men experiencing ED do not seek medical attention and that several barriers for healthcare seeking with ED are present [9]. Common barriers for healthcare seeking with ED reported in the literature are infrequent occurrence or lack of importance for the individual [10]. Hesitating to consult the GP seems reasonable when ED is a passing issue and if the individual is not bothered by ED. However, little is known about healthcare-seeking and barriers for the group of men who are bothered by ED. This could be valuable knowledge because these men might benefit from consulting their GP for evaluation and effective treatment. Healthcare-seeking regarding intimate symptoms has been shown to vary between socioeconomic groups [11] and patients with ED who have low household income and educational level have longer help seeking intervals [12] It is plausible that these differences in healthcare seeking behavior could be due to differences in barriers for healthcare seeking and it has been shown that barriers for healthcare seeking with ED differ between age groups [10]. However, little is known about the association between socioeconomic characteristics of the patient and barriers for healthcare seeking with bothersome ED. Our hypotheses are that elder men are less likely to contact their GP when bothered by ED and that that younger men with lower socioeconomic status are more likely to report barriers for GP contact.

The objectives of this population-based study are thus 1) to estimate the proportion of men bothered by ED, who do not contact the GP, 2) to analyse the frequencies of different barriers for healthcare seeking and 3) to analyse associations between socioeconomic factors and barriers for contacting the GP.

## Methods

### Sampling procedure

Data for this study derive from a nationwide survey of symptom experiences among 100,000 individuals aged 20 years and above randomly selected from the Danish Civil Registration System which comprises contact information for all Danish citizens [13]. The 100,000 individuals were invited by letter to participate in the online survey via a secure web page. Telephone interviews were offered to those without Internet access. Invited individuals not responding were reminded by a letter and afterwards by telephone. Reasons for non-participation were registered if stated. The data collection took place from June to December 2012. Details about the study sample is described elsewhere [14].

### Questionnaire

For the survey a comprehensive questionnaire about various symptom experiences, including ED, was developed. The questionnaire was pilot- and field-tested prior to distribution [14]. When a respondent confirmed to have experienced the symptom, a number of follow-up questions were asked about the symptom’s influence on daily activities, concerns about the symptom, whether the respondent had contacted the GP regarding the symptom and considerations about healthcare seeking with the symptom.

The questions about ED form the basis of this study. The question about experiencing ED was phrased: “Have you within the preceding four weeks experienced erectile dysfunction?” In addition to confirming or denying the presence of ED, the respondents had the possibility to reply, “Do not wish to answer”. Respondents confirming to have experienced ED were subsequently asked if they had contacted their GP about the symptom. Furthermore, the respondents were asked to report to what extent they were concerned about ED and to what extent ED interfered with their usual daily activities. For this purpose, a five-point Likert scale with the options: “not at all”, “slightly”, “moderate”, “quite a bit” and “extremely”, was used. Additionally, the respondents were asked if they had had any considerations about contacting their GP. The respondents could choose between four predefined barriers and an “other considerations” category. The wording of the barriers was: “I would be too embarrassed to go to the doctor”, “I would be worried about wasting the doctor’s time”, “I was too busy to make time to go to the doctor”, “I would be worried about what the doctor might find”.

This study includes all men experiencing ED with the primary focus on the group who reported bothersome ED and further reported, that they did not contact a GP regarding the symptom. The term bothersome covers ED which was reported as either moderately to extremely concerning and/or moderately to extremely influencing on daily activities.

### Socioeconomic data

Information about income, education, labour market affiliation and cohabitation status was obtained from Statistics Denmark [15–17]. Each Danish citizen is assigned a unique personal identification number enabling accurate linkage between registers. Highest attained educational level at the time of filling in the questionnaire was divided into three groups < 10 years, 10–14 years and ≥ 15 years of school.

Average disposable income was defined as the entire household income after taxation, adjusted for number of persons in the household in the year of filling in the questionnaire. Disposable income was divided into low (first quartile), medium (second and third quartile) and high (fourth quartile). Labour market affiliation was categorized as working, retired or out of workforce according to the status each respondent predominantly had in the year of filling in the questionnaire. Out of workforce comprises disability pension and unemployment. Cohabitation status at the time of participating in the survey was categorized as married/cohabiting or living alone.

### Statistical analysis

The proportions of men with bothersome and not bothersome ED, respectively, and the proportion of men not consulting the GP in each group were calculated. Moreover, age distribution and socioeconomic characteristics are described for each group. Socioeconomic characteristics were defined by the following co-variates: age group, marital status, educational level, labor market affiliation, income and ethnicity. The primary outcome was defined as men with bothersome ED who did not contact the GP. Possible associations between not contacting the GP with bothersome ED and the co-variates were assessed as odds ratios (ORs) using multiple logistic regression. Crude and adjusted odds ratios with 95% confidence intervals (CI) were calculated.

Further, six binary outcome variables were defined representing the five predefined barriers and the category ‘none’. The proportion of reported barriers towards GP contact are presented according to age and socioeconomic characteristics among men with bothersome ED who did not contact the GP.

Finally, multiple logistic regression models were used to calculate crude and adjusted ORs for associations between reported barriers towards GP contact among men who did not contact the GP with bothersome ED and socioeconomic characteristics.

Data analyses were conducted using STATA statistical software (StataCorp, College Station, TX, USA). All tests used a significance level of *p* < 0.05.

## Results

**Fig. 1:**
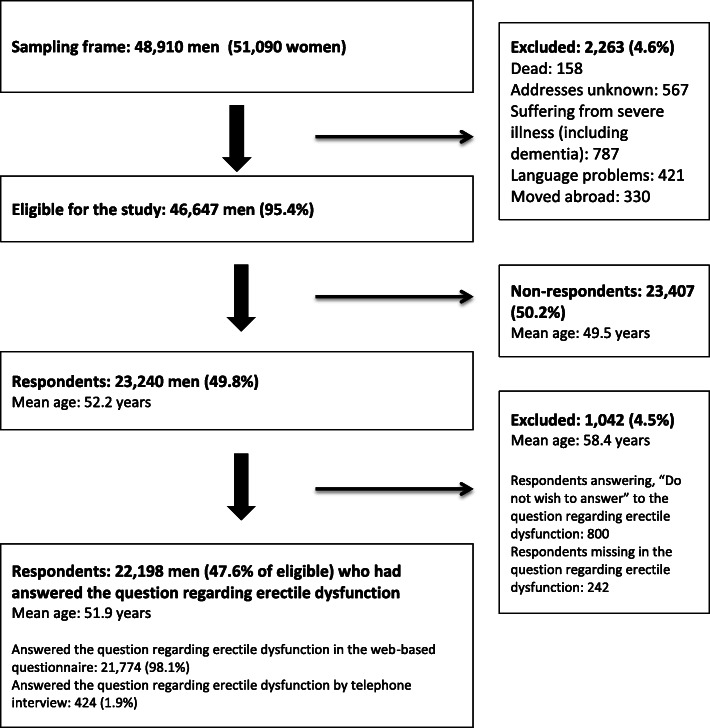
Study Cohort, 100,000 randomly selected Danish adults + 20 years

A total of 48,910 Danish men were invited to participate in the survey. 2263 men (4.6%) were ineligible for the study since they had either died, could not be reached due to unknown addresses, had severe illness, had language problems, or had moved abroad. A total of 23,240 (49.8%) completed the questionnaire, and of these 1042 (4.5% of respondents) did not wish to answer or were missing in the question regarding ED, resulting in a study cohort of 22,198 men (47.6%) (Fig. 1).

A total of 4072 men (18.3%) reported that they had experienced ED within the past four weeks. Of those, 2888 (70.9%) were categorized as having bothersome ED. In the group of men with bothersome ED 1802 (62.4%) did not contact the GP regarding their ED (Table 1).

**Table 1:** Socioeconomic characteristics for men with erectile dysfunction

	Bothersome^b^ ED		ED, but not bothersome	
	N %	Did not contact the GP, %	N %	Did not contact the GP, %
All	2888 (100.0)	1802 (62.4)	1184 (100.0)	951 (80.3)
**Age**				
20–39	77 (2.7)	60 (77.9)	45 (3.8)	35 (77.8)
40–59	735 (25.5)	467 (63.5)	233 (19.7)	183 (78.5)
60–79	1889 (65.4)	1143 (60.5)	789 (66.6)	628 (79.6)
80+	187 (6.5)	132 (70.6)	117 (9.9)	105 (89.7)
Socioeconomic status				
**Marital status**				
Single	489 (16.9)	300 (61.3)	250 (21.1)	192 (76.8)
Married/Cohabiting	2399 (83.1)	1502 (62.6)	934 (78.9)	759 (81.3)
**Educational level**				
Low (< 10 years)	542 (18.8)	331 (61.1)	206 (17.4)	164 (79.6)
Middle (10–14 years)	1579 (54.7)	1010 (64.0)	591 (49.9)	471 (79.7)
High (≥15 years)	767 (26.6)	461 (60.1)	387 (32.7)	316 (81.7)
**Labour market affiliation**				
Working	1169 (40.5)	754 (64.5)	453 (38.3)	362 (79.9)
Retirement pension	1465 (50.7)	902 (61.6)	666 (56.3)	538 (80.8)
Out of workforce^a^	254 (8.8)	146 (57.5)	65 (5.5)	51 (78.5)
**Equivalence weighted disposable income**				
Low (1st quartile)	471 (16.3)	302 (64.1)	216 (18.2)	167 (77.3)
Middle (2nd and 3rd quartile)	1535 (53.2)	930 (60.6)	603 (50.9)	493 (81.8)
High (4th quartile)	882 (30.5)	570 (64.6)	365 (30.8)	291 (79.7)
**Ethnicity**				
Danish	2774 (96.1)	1730 (62.4)	1138 (96.1)	915 (80.4)
Immigrants and descendants of immigrants	114 (3.9)	72 (63.2)	46 (3.9)	36 (78.3)

^a^Comprises disability pension and unemployment

^b^Bothersome ED is defined by influencing daily activities and/or concern for having ED

Men aged 40–79 years and men out of workforce had lower odds of not contacting the GP with bothersome ED (Table 2).

**Table 2:** Associations between not contacting the GP regarding bothersome erectile dysfunction and socioeconomic status

	Crude OR (95% CI)	Adj OR (95% CI)^a^
**Age**		
20–39	1	1
40–59	0.49 (0.28–0.86)	**0.47 (0.27–0.83)**
60–79	0.43 (0.25–0.75)	**0.42 (0.24–0.73)**
80+	0.68 (0.36–1.27)	0.67 (0.36–1.26)
**Marital status**		
Single	1	1
Married/Cohabiting	1.05 (0.86–1.29)	1.13 (0.92–1.38)
**Educational level**		
Low (< 10 years)	1	1
Middle (10–14 years)	1.13 (0.93–1.38)	1.11 (0.91–1.36)
High (≥15 years)	0.96 (0.77–1.20)	0.95 (0.75–1.19)
**Labour market affiliation**		
Working	1	1
Retirement pension	0.88 (0.75–1.03)	0.92 (0.75–1.13)
Out of workforce^b^	0.74 (0.56–0.98)	**0.75 (0.57–0.99)**
**Equivalence weighted disposable income**		
Low (1st quartile)	1	1
Middle (2nd and 3rd quartile)	0.86 (0.69–1.07)	0.86 (0.69–1.07)
High (4th quartile)	1.02 (0.81–1.29)	1.08 (0.83–1.39)
**Ethnicity**		
Danish	1	1
Immigrants and descendants of immigrants	1.03 (0.70–1.53)	1.00 (0.68–1.48)

^a^Adjustments were made for all other covariates

^b^Comprises disability pension and unemployment

A total of 60.5% of the men with bothersome ED and no GP contact reported barriers for GP contact. The most frequent barrier was ‘being too embarrassed’ reported by 29.7%. The second most common barrier was ‘other’ reported by 20.8%. Among those with any barrier, 49.0% reported embarrassment (Table 3).

**Table 3:** Reported barriers towards GP contact in numbers (n) and proportion according to symptom, age among men who did not contact the GP

	Total	Being too embarrassed	Wasting the GP’s time	Worried about what the GP might find	Being too busy	Other	None
ED influence and/or concern and did not contact the GP	N = (100%)	N (%)	N (%)	N (%)	N (%)	N (%)	N (%)
	1802 (100.0%)	535 (29.7%)	257 (14.3%)	226 (12.5%)	207 (11.5%)	374 (20.8%))	711 (39.5%)
**Age**							
20–39	60 (3.3%)	31 (61.7%)	12 (20.0%)	17 (28.3%)	18 (30.0%)	10 (16.7%)	12 (20.0%)
40–59	467 (25.9%)	173 (37.0%)	70 (15.0%)	86 (18.4%)	82 (17.6%)	89 (19.1%)	142 (30.4%)
60–79	1143 (63.4%)	290 (25.4%)	154 (13.5%)	104 (9.1%)	96 (8.4%)	251 (22.0%)	496 (43.4%)
80+	132 (7.3%)	41 (31.1%)	21 (15.9%)	19 (14.4%)	11 (8.3%)	24 (18.2%)	61 (46.2%)
**Marital status**							
Single	300 (16.6%)	89 (29.7%)	48 (16.0%)	44 (14.7%)	36 (12.0%)	76 (25.3%)	103 (34.3%)
Married/Cohabiting	1502 (83.4%)	446 (29.7%)	209 (13.9%)	182 (12.1%)	171 (11.4%)	298 (19.8%)	608 (40.5%)
**Educational level**							
Low (< 10 years)	331 (18.4%)	110 (33.2%)	57 (17.2%)	43 (13.0%)	29 (8.8%)	43 (13.0%)	148 (44.7%)
Middle (10–14 years)	1010 (56.0%)	307 (30.4%)	142 (14.1%)	141 (14.0%)	128 (12.7%)	187 (18.5%)	400 (39.6%)
High (≥15 years)	461 (25.6%)	118 (25.6%)	58 (12.6%)	42 (9.1%)	50 (10.8%)	144 (31.2%)	163 (35.6%)
**Labour market affiliation**							
Working	754 (41.8%)	233 (30.9%)	108 (14.3%)	105 (13.9%)	130 (17.2%)	167 (22.1%)	253 (33.6%)
Retirement pension	902 (50.1%)	246 (27.3%)	124 (13.7%)	85 (9.4%)	65 (7.2%)	179 (19.8%)	417 (46.2%)
Out of workforce^a^	146 (8.1%)	56 (38.4%)	25 (17.1%)	36 (24.7%)	12 (8.2%)	28 (19.2%)	41 (28.1%)
**Equivalence weighted disposable income**							
Low (1st quartile)	302 (16.8%)	91 (30.1%)	47 (15.6%)	43 (14.2%)	35 (11.6%)	57 (18.9%)	119 (39.4%)
Middle (2nd and 3rd quartile)	930 (51.6%)	283 (30.4%)	134 (14.4%)	121 (13.0%)	102 (11.0%)	172 (18.5%)	385 (41.4%)
High (4th quartile)	570 (31.6%)	161 (28.2%)	76 (13.3%)	62 (10.9%)	70 (12.3%)	145 (25.4%)	207 (61.8%)
**Ethnicity**							
Danish	1730 (96.0%)	516 (29.8%)	244 (14.1%)	213 (12.3%)	190 (11.0%)	357 (20.6%)	694 (40.1%)
Immigrants and descendants of immigrants	72 (4.0%)	19 (26.4%)	13 (18.1%)	13 (18.1%)	17 (23.6%)	17 (23.6%)	17 (23.6%)

^a^Comprises disability pension and unemployment

For ‘being too embarrassed’, ‘worried about what the doctor might find’ and ‘being too busy’ odds of reporting these barriers were lower in the older age groups, although not significantly for the age group 40–59 years regarding the barrier ‘worried about what the doctor might find’ (Table 4).

**Table 4:** Associations between age, Socioeconomic status and reported barriers for GP contact regarding ED

Bothersome ED	Being too embarrassed		Wasting the GP’s time		Worried about what the GP might find		Being too busy		Other		None	
	Crude OR (95% CI)	Adj. OR^a^ (95% CI)	Crude OR (95% CI)	Adj. OR^a^ (95% CI)	Crude OR (95% CI)	Adj. OR^b^ (95% CI)	Crude OR (95% CI)	Adj. OR^b^ (95% CI)	Crude OR (95% CI)	Adj. OR^b^ (95% CI)	Crude OR (95% CI)	Adj. OR^b^ (95% CI)
**Age**												
20–39	1	1	1	1	1	1	1	1	1	1	1	1
40–59	0.55 (0.32–0.94)	**0.51 (0.29–0.89)**	0.71 (0.36–1.39)	0.72 (0.36–1.45)	0.57 (0.31–1.05)	0.56 (0.30–1.05)	0.50 (0.27–0.91)	**0.47 (0.25–0.87)**	1.18 (0.57–2.41)	1.43 (0.69–2.97)	1.75 (0.90–3.39)	1.61 (0.82–3.14)
60–79	0.32 (0.19–0.54)	**0.29 (0.17–0.50)**	0.62 (0.32–1.20)	0.63 (0.32–1.24)	0.25 (0.14–0.46)	**0.25 (0.14–0.47)**	0.21 (0.12–0.39)	**0.21 (0.11–0.38)**	1.41 (0.70–2.81)	1.70 (0.84–3.47)	3.07 (1.61–5.83)	2.83 (1.47–5.44)
80+	0.42 (0.23–0.79)	**0.39 (0.21–0.74)**	0.76 (0.34–1.66)	0.76 (0.34–1.69)	0.43 (0.20–0.89)	**0.43 (0.20–0.92)**	0.21 (0.09–0.49)	**0.21 (0.09–0.48)**	1.11 (0.49–2.50)	1.24 (0.54–2.82)	3.44 (1.67–7.05)	3.27 (1.58–6.75)
**Marital status**												
Single	1	1	1	1	1	1	1	1	1	1	1	1
Married/Cohabiting	1.00 (0.76–1.31)	1.17 (0.88–1.55)	0.85 (0.60–1.19)	0.90 (0.63–1.28)	0.80 (0.56–1.15)	0.96 (0.66–1.40)	0.94 (0.64–1.38)	1.16 (0.77–1.74)	0.73 (0.55–0.97)	0.69 (0.51–0.93)	1.30 (1.00–1.69)	1.19 (0.91–1.56)
**Educational level**												
Low (< 10 years)	1	1	1	1	1	1	1	1	1	1	1	1
Middle (10–14 years)	0.88 (0.67–1.15)	0.78 (0.59–1.02)	0.79 (0.56–1.10)	0.77 (0.55–1.08)	1.09 (0.75–1.57)	0.93 (0.64–1.36)	1.51 (0.99–2.31)	1.26 (0.82–1.94)	1.52 (1.06–2.18)	**1.59 (1.11–2.28)**	0.81 (0.63–1.04)	0.90 (0.69–1.16)
High (≥ 15 years)	0.69 (0.51–0.94)	**0.65 (0.48–0.90)**	0.69 (0.47–1.03)	0.68 (0.46–1.01)	0.67 (0.43–1.05)	**0.62 (0.40–0.98)**	1.27 (0.78–2.05)	1.18 (0.72–1.92)	3.04 (2.09–4.43)	**3.10 (2.13–4.53)**	0.68 (0.51–0.90)	0.70 (0.52–0.93)
**Labour market affiliation**												
Working	1	1	1	1	1	1	1	1	1	1	1	1
Retirement pension	0.84 (0.68–1.04)	1.26 (0.93–1.70)	0.95 (0.72–1.26)	0.96 (0.66–1.40)	0.64 (0.47–0.87)	0.93 (0.60–1.43)	0.37 (0.27–0.51)	0.55 (0.36–0.84)	0.87 (0.69–1.10)	0.76 (0.56–1.03)	1.70 (1.39–2.08)	1.35 (1.04–1.75)
Out of workforce^a^	1.39 (0.96–2.01)	1.38 (0.94–2.01)	1.23 (0.77–1.99)	1.16 (0.72–1.89)	2.02 (1.32–3.10)	**1.97 (1.27–3.07)**	0.43 (0.23–0.80)	**0.43 (0.23–0.81)**	0.83 (0.53–1.30)	0.88 (0.56–1.40)	0.77 (0.52–1.14)	0.77 (0.51–1.14)
**Equivalence weighted disposable income**												
Low (1st quartile)	1	1	1	1	1	1	1	1	1	1	1	1
Middle (2nd and 3rd quartile)	1.01 (0.76–1.35)	1.01 (0.75–1.36)	0.91 (0.64–1.31)	0.97 (0.67–1.41)	0.90 (0.62–1.31)	0.90 (0.60–1.33)	0.94 (0.62–1.41)	0.89 (0.58–1.38)	0.98 (0.70–1.36)	0.99 (0.70–1.40)	1.09 (0.83–1.42)	1.09 (0.83–1.44)
High (4th quartile)	0.91 (0.67–1.24)	0.95 (0.68–1.33)	0.84 (0.56–1.24)	0.96 (0.62–1.47)	0.74 (0.49–1.12)	0.76 (0.48–1.21)	1.07 (0.69–1.65)	0.95 (0.59–1.54)	1.47 (1.04–2.07)	1.26 (0.86–1.84)	0.88 (0.66–1.17)	0.97 (0.71–1.33)
**Ethnicity**												
Danish	1	1	1	1	1	1	1	1	1	1	1	1
Immigrants and descendants of immigrants	0.84 (0.49–1.44)	0.73 (0.42–1.26)	1.34 (0.72–2.48)	1.34 (0.72–2.50)	1.57 (0.85–2.91)	1.31 (0.69–2.49)	2.51 (1.42–4.41)	**1.95 (1.09–3.49)**	1.19 (0.68–2.07)	1.08 (0.61–1.90)	0.46 (0.27–0.80)	0.57 (0.32–1.00)

^a^ Comprises disability pension and unemployment, ^b^Adjustments were made for marital status, age group and educational level

Respondents with high educational level had lower odds of reporting ‘being too embarrassed’ and ‘worried about what the doctor might find’ and higher odds of reporting ‘other’ as a barrier. Respondents with middle educational level also had higher odds of reporting ‘other’ as a barrier (Table 4).

The respondents who were out of workforce had higher odds of reporting ‘worried about what the doctor might find’ OR 1.97(95% CI 1.27–3.07) and lower odds of ‘being too busy’ OR 0.43(95% CI 0.23–0.81).

Respondents with non-Danish ethnic backgrounds had higher odds of reporting ‘being too busy’ OR 1.95 (95% CI 1.09–3.49).

## Discussion

### Main findings

Most of the men (70.9%) reporting ED was categorized as having bothersome ED and the majority of those (62.4%) did not contact the GP. Among those bothered by ED and not contacting their GP, 60.6% reported barriers for GP contact. The most common barrier was ‘being too embarrassed’ (29.7%).

In general, respondents in the older age groups were less likely to report embarrassment, business and worrying what the GP might find as a barrier. Respondents with highest attained educational level were less likely to have embarrassment and worrying as barriers for not contacting the GP, but more often indicated other considerations. The respondents who were out of work force were more likely to be worried about what the doctor might find.

### Strengths and weaknesses of the study

This study included a large number of randomly selected individuals’ representative of the Danish adult population. The overall response rate among men was 49.8%. The respondents were slightly older compared to the non-respondents (Fig. 1).

A total of 1042 men either missed or did not wish to answer the question concerning ED resulting in a response rate of 47.6% for the ED question, which is comparable to similar studies covering self-reported ED [18–20]. Individuals with missing information or not wishing to answer the ED question were generally older (Fig. 1). Some individuals might consider ED as a topic too intimate to report. Not wishing to answer a question regarding ED could hypothetically be associated to barriers for contacting the GP, leading to an underestimation of the proportion of men not consulting the GP and hence also underreporting of some of the five barriers.

Our design using a web-based questionnaire is an advantage as it provides anonymity. Participants completing the questionnaire by telephone interview might find the topic too delicate and tend to dismiss their ED compared to those completing the web-based questionnaire. However, only 1.9% of the respondents completed the question regarding ED by telephone interview (Fig. 1) and a possible difference is therefore presumed to minimally influence the results.

The understanding and interpretation of the question regarding ED might depend on age, cohabitation status, sexual activity etc. However, the field and pilot testing did not reveal problems in relation to this. Participants were asked to recall symptom experience during the preceding four weeks. The short recall period reduces risk of recall error. As we addressed bothersome ED i.e. which either worried or influenced daily activities, it seems reasonable to assume a correct recall within this time frame.

The definition of bothersome ED was constructed by two questions regarding worrying about having ED and the degree of influence on daily activity. It is possible that some respondents have answered negatively on these two questions but still feeling bothered by their ED.

The questionnaire did not contain information on for how long the respondents had been bothered by ED. It only states whether they had experienced ED within the past four weeks. Therefore, it might seem reasonable not to contact the GP if they had only experienced ED once.

In addition to the predefined barriers the questionnaire also comprised an open-ended category with the possibility to express other barriers. These statements are gathered in an ‘other’ category in the present study. Qualitatively exploring the statements in the ‘other’ category is beyond the scope of this study.

### Comparison with existing literature

In a survey of male health issues from 2000 conducted in six western countries (US and Europe) *Shabsigh* et al found that 53.0% of men aged 20–75 years experiencing ED had not sought treatment [10], which is slightly lower than our findings. This difference could be due to this study including men > 75 years as well or by the fact that *Shabsigh* et al recruited respondents visiting their physician, i.e. their respondents might have a higher tendency to contact their GP in general and therefore be more likely to seek medical attention when experiencing ED as well.

Further, *Shabsigh* et al found that younger men were less likely to contact the GP regarding ED, which is comparable to our findings. They found that reasons for not seeking medical attention differed between age groups as the younger men believed that their ED would resolve spontaneously, whereas older men thought that ED was a natural part of ageing. We did not measure such considerations in our study but that may explain the low proportion of health seeking among the youngest and oldest respondents in our study and may be the reasons for the frequent choosing of the barrier ‘other’.

In a Turkish survey of men with ED *Gulpinar* et al [12] found that embarrassment was the most frequent reason for delayed consultation underlining our result of embarrassment being an important barrier for GP contact. The ability to perform sexually is linked to the male masculine role and societal expectations, hence problems relating to this is, although very common, still a taboo.

To our knowledge no studies have previously studied ‘being too busy’ and ‘worrying what the doctor might find’ as barriers for healthcare-seeking with ED. Odds of reporting these barriers were lower in the older age groups. As it becomes more likely that ED often is due to a structural disease with increasing age, it is a reassuring finding that worrying what the doctor might find, is not considered as a barrier for older men. It is expectable that increasing age is not associated with being too busy as older men are not as often congested by e.g. work.

### Implications

The fact that most respondents bothered by ED did not contact their GP and reported embarrassment as a frequent barrier highlights the importance of the GP taking a proactive approach to identifying patients bothered by ED and breaking down the taboo of ED. However, several other barriers exist for dealing sufficiently with sexual health issues in general practice such as constraints of time and expertise [21]. It has been shown that patients are more likely to seek help regarding sexual health if their doctor had asked about sexual function during a routine visit sometime during the previous years [22] so it might not be unrealistically time-consuming to reduce the patients’ hesitation to consult when being bothered by ED. Therefore, it could be an idea to implement it as a standard subject in routine consultations for hypertension etc.

## Conclusion

Our study shows that nearly two third of the respondents bothered by ED did not contact their GP regarding ED. More than half of the men who did not contact the GP reported barriers towards GP contact. Being too embarrassed to go to the doctor was the most common barrier. Older respondents were more likely than young men to contact their GP regarding ED and less likely to report embarrassment, business and worrying what the GP might find as a barrier. The respondents with highest attained educational level were less likely to report embarrassment and worrying as barriers for not contacting the GP.

## Supplementary information

**Additional file 1.**

## Data Availability

The datasets generated and analysed during the current study are not publicly available due to the data protection regulations of the Danish Data Protection, Statistics Denmark and the Danish Health and Medicines Authority. Access to data is strictly limited to the researchers who have obtained permission for data processing. This permission was giving to the Research Unit of General Practice, Department of Public Health, University of Southern Denmark.

## Abbreviations

GP: General Practitioner
ED: Erectile Dysfunction
