# Supplementary material for: Socioeconomic status and barriers for contacting the general practitioner when bothered by erectile dysfunction: a population-based cross-sectional study
Source: BMC Fam Pract. 2020 Aug 16;21:166. doi: 10.1186/s12875-020-01238-2 (PMC7429708; doi:10.1186/s12875-020-01238-2)
Supplement: Supplementary file 1 — Additional file 1. [file 12875_2020_1238_MOESM1_ESM.docx]

The next question may seem private, but your response may contribute to a greater understanding of the prevalence of symptoms or discomfort in the population. If you do not wish to answer the question, simply tick the category "Do not wish to answer."

*Only for men:*

Have you within **the last 4 weeks** experienced any of the following?

- Erectile dysfunction
- Blood in the semen
- None of the above
- I don’t wish to answer

*The following questions only appeared in relation to a positive expression of one or more experienced symptom(s) - by a leap structure in the electronic survey to the symptom experience*

| We will now ask you some questions concerning who you have talked to about the symptoms or discomfort you experienced **in the last 4 weeks**. |
| --- |

Have you contacted your general practitioner with any of the following symptoms or discomfort? (Through appointment, by telephone or by email)

- Yes
- No

*The following questions only appeared in relation to a positive expression of one or more experienced symptom(s) - by a leap structure in the electronic survey to the symptom experience*

| You have ***not*** been in contact with your general practitioner regarding the following symptoms and discomforts. We would now like to know, whether you had some of the following considerations, regarding contact to your general practitioner? (You may tick more than one box) |
| --- |

|  |  | Yes | No |
| --- | --- | --- | --- |
| Erectile dysfunction  Etc. | I would be too embarrassed |  |  |
|  | I would be worried about wasting the doctor’s time |  |  |
|  | I would be worried about what the doctor might find |  |  |
|  | I would be too busy to make time to go to the doctor |  |  |
|  | Other considerations [box for free text commentaries] |  | |
